# Circulating IL-6, clusterin and irisin in obese subjects with different grades of obesity: association with insulin resistance and sexual dimorphism

**DOI:** 10.20945/2359-3997000000336

**Published:** 2021-02-25

**Authors:** Rehab H. Werida, Nashwa M. El-Gharbawy, Tarek M. Mostafa

**Affiliations:** 1 Damanhur University Faculty of Pharmacy Department of Clinical Pharmacy and Pharmacy Practice Damanhur Egypt Department of Clinical Pharmacy and Pharmacy Practice, Faculty of Pharmacy, Damanhur University, Damanhur, Egypt.; 2 Tanta University Faculty of Medicine Diabetes & Endocrinology Unit Tanta Egypt Department of Internal Medicine, Diabetes & Endocrinology Unit, Faculty of Medicine, Tanta University, Tanta, Egypt.; 3 Tanta University Faculty of Pharmacy Department of Clinical Pharmacy Tanta Egypt Department of Clinical Pharmacy, Faculty of Pharmacy, Tanta University, Tanta, Egypt.

**Keywords:** IL-6, clusterin, irisin, HOMA-IR, obesity

## Abstract

**Objective:**

There are discrepancies about the relationship of IL-6, clusterin and irisin with obesity and obesity associated insulin resistance and also about their sexual dimorphism. This study aimed at evaluating the circulating levels of IL-6, clusterin and irisin in obese subjects of both sexes who had different grades of obesity and examining their sexual dimorphism and their association with insulin resistance.

**Subjects and methods:**

This study included 176 non-diabetic subjects of both sexes who were classified according to their sex into two groups; the male and the female groups. The male group (88 men) was classified according to BMI into; group 1 (22 lean men), group 2 (22 class I obese men), group 3 (22 class II obese men) and group 4 (22 class III obese men). The female group (88 women) was classified according to BMI exactly as the male group. Metabolic parameters, IL-6, clusterin, and irisin levels were measured. Data were analyzed by ANOVA test, post hoc Tukey’s test and independent t-test. Pearson correlation was used to assess the association between variables.

**Results:**

In obese subjects of both sexes, circulating IL-6, clusterin and irisin levels were significantly elevated and positively correlated with HOMA-IR. Obese males showed significantly higher HOMA-IR, IL-6, clusterin and irisin levels than obese females.

**Conclusion:**

Obesity in both sexes, especially in males was associated with high levels of IL-6, clusterin and irisin and worsened the metabolic pattern. Circulating IL-6, clusterin and irisin may represent possible therapeutic targets for insulin resistance in obese subjects.

## INTRODUCTION

Obesity is a major health problem secondary to its related complications including insulin resistance, dyslipidemia and cardiovascular diseases. Chronic inflammation particularly in adipose tissues and liver links obesity to insulin resistance and even to the development of type 2 diabetes mellitus (1). Although, chronic inflammation remains the main mechanism triggers insulin resistance, adipose tissue produces both pro- and anti-inflammatory substances including IL-6, clusterin and irisin which may play a role in glucose hemostasis (2,3). However, the major source of IL-6 is the adipose tissue; Il-6 is also produced by muscles and many cell types including fibroblasts, endothelial cells, mast cells and immune cells (4-6). Clusterin was reported to be secreted and expressed by adipocytes, hepatocytes, brain, ovary, testis, heart, and blood vessels (3,7). Irisin was suggested to be released from skeletal muscles and adipocytes (2).

Il-6, the major inflammatory mediator was reported to be positively associated with adiposity and insulin resistance (4,8). Clusterin is a multifunctional, stress-induced, ATP-independent molecular chaperone (9). Clusterin plays a vital role in lipid transportation, inflammation and immune responses (10). Also, it was postulated that, circulating clusterin level was associated with adiposity and inflammation (3,11). Irisin is not only a myokine but also an adipokine (12). Irisin was reported to increase total energy expenditure, improve glucose tolerance and reduce fasting insulin level; therefore it improves glucose homeostasis, insulin resistance and obesity-related health conditions (2).

IL-6 is regulated by several factors including hormones, cytokines, stress, hypoxia, infection excercise, and cold exposure (4,13,14). Clusterin exists in two forms; the nuclear form and the secreted extracellular clusterin (9). The secreted form is upregulated as a defense mechanism during oxidative stress (9). Hepatic clusterin mRNA expression and serum clusterin level have been shown to be remarkably elevated after exposure to inflammatory cytokines (15). Also, smoking may contribute to increased circulating clusterin level (11). It was reported that, irisin level tends to be increased with acute exercise and remained unchanged with chronic exercise (16).

There are discrepancies about the relationship of both clusterin and irisin levels with adiposity and obesity associated insulin resistance (2,3,11,17-20). In addition, the effect of both female and male sex hormones on both clusterin and irisin levels seems inconsistent (11,21-23). Furthermore, the previous studies aimed at evaluating the levels of clusterin and irisin in lean, overweight and obese subjects with little respect to grades of obesity (11,19). Therefore, our study aimed at evaluating the circulating levels of IL-6, clusterin and irisin in obese subjects of both sexes who had different grades of obesity and examining their sexual dimorphism and their association with insulin resistance.

## SUBJECTS AND METHODS

### Study design and population

In this observational parallel study, 220 subjects were selected as a convenience sample from consecutive admission to outpatient clinic of Diabetes & Endocrinology Unit, Internal Medicine Department, Tanta University Hospital, Tanta, Egypt, between February 2018 and June 2019 when they fulfilled the study inclusion criteria which included adult, age matched (age range 24-38 years old), both sexes, variable BMI, non-smokers and non-diabetic subjects (fasting blood glucose ≤126 mg/dL). Out of those 220 subjects selected, 35 subjects were excluded according to the study exclusion criteria. The remaining 185 subjects were enrolled in the study and were classified according to their sex into two main groups; the male group (n = 95) and the female group (n = 90). The male group (95 men) was subdivided according to BMI into; lean male group (n = 24), class I obese male group (n = 25), class II obese male group (n = 24), and class III obese male group (n = 22). The female group (90 women) was sub-classified according to BMI into; lean female group (n = 22), class I obese female group (n = 23), class II obese female group (n = 23) and class III obese female group (n = 22). Seven subjects in the male group and 2 subjects in the female group were dropped out due to their decline to participate. The final analysis included 176 subjects (88 subjects in each group with 22 subjects in each BMI class). The exclusion criteria included subjects with history of diabetes mellitus, liver, renal, thyroid, inflammatory diseases, and females on contraceptives pills. Patients on medications that can interfere with glucose or lipid metabolism (hypoglycemic agents, corticosteroids, anti-hyperlipidemics, non-selective beta blockers, thiazides, etc.) and subjects with organic causes of obesity were also excluded. The protocol of this study was approved by the National Research Ethics Committee of Tanta University, Egypt. This study was registered on ClinicalTrials.gov with ID no. NCT04133896. Eligible patients gave their written informed consent. All individuals included in the study were submitted to medical history (past or current illness), demography (age, diet supplement), and measurements of weight, height and waist circumference. Waist circumference was measured at the approximate midpoint between the inferior costal margin and the superior border of the iliac crest with non-elastic flexible tape. Waist/height ratio and body mass index (BMI) were calculated. BMI is defined as the weight in kilograms divided by the square of the height in meters; BMI = [Weight (kg) /Height^2^ (m)]. The subjects enrolled in the study were distributed in different groups according to the criteria of The National Institute for Health and Clinical Excellence (NICE 2006) which classifies obesity according to the BMI as follows; healthy weight or normal weight (BMI: 18.5–24.9 kg/m^2^), overweight (BMI: 25–29.9 kg/m^2^), class I obesity (BMI: 30–34.9 kg/m^2^), class II obesity (BMI: 35–39.9 kg/m^2^), and class III obesity (BMI: ≥40 kg/m^2^). All subjects were submitted to blood sample collection before starting any medications, dietary or exercise programs. In addition, all participants were instructed to abstain from physical activity 24 hours before blood sample collection to avoid interference with IL-6 and irisin assay.

### Laboratory analysis

#### Sample collection

Ten ml of venous blood was collected from all participants (between 8-10 am) after an overnight (12 hour) fasting by venipuncture of the antecubital vein into sterile tubes. Plasma and sera were separated and were stored at -80 °C until biochemical analysis of fasting insulin, IL-6, clusterin and irisin levels. Fresh samples were used to evaluate lipid profile and fasting blood glucose.

#### Assay

Plasma triglycerides and total cholesterol were measured by enzymatic colorimetric method. High-density lipoprotein cholesterol (HDL-C) was measured by precipitation method. Low density lipoprotein cholesterol (LDL-C) was calculated using the Friedewald formula:

LDL = [TC – HDL - (TG/5)] provided that TG level was less than 400 mg/dL. Fasting blood glucose level was assayed by glucose oxidase method. Fasting plasma insulin (FPI) was assayed using Enzyme-Linked Immunosorbent Assay kit based on the sandwich principle (DRG international, Inc., USA: Reference No.: EIA-2935). We estimated insulin resistance (IR) using the HOMA-IR index which is defined as fasting insulin level (μIU/mL) times fasting blood glucose (mmol/L) divided by 22.5 or divided by 405 if fasting blood glucose is expressed in mass units (mg/dL). Quantitative determination of circulating IL-6, clusterin and irisin levels were done using the commercially available ELISA kits (Sun Red Biotechnology Company, Shanghai, China: Catalogue No.: 201-12-0091; 201-12-1190 and 201-12-5328 respectively).

### Statistical analysis

Data were statistically analyzed by SPSS software for Windows version 25 (Chicago, IL, USA). Data were analyzed by One-way analysis of variance test followed by post hock Tukey’s HDS test for multiple comparisons within the male and the female groups. Independent *t*-test was used to compare the male and the female groups. Correlations between variables were assessed using Pearson correlation analysis. All results were expressed as mean ± SD. The significance level was set at (p<0.05).

## RESULTS

**Figure 1:**
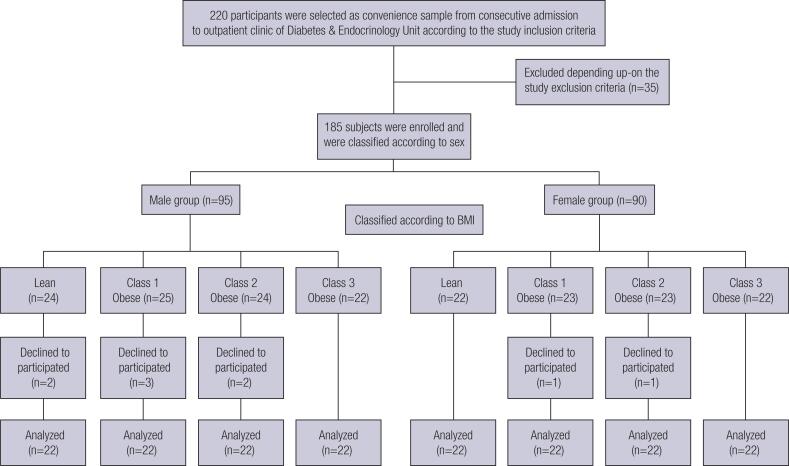
Participants screening and enrollment flow chart.

The study participants and their selection and classification according to their sex and BMI are illustrated in Figure 1. The anthropometric data and biological parameters in the different studied male groups were shown in Table 1. As compared to lean male group, class I, II and III obese male groups showed significant elevation in all measured variables (p=0.000) except for HDL-C level which showed significant decease (p=0.000).

**Table 1:** Anthropometric data and biochemical parameters in the different studied male groups

Parameters	Lean group (n=22)	Class I obese group (n=22)	P-value Class I vs Lean	Class II obese group (n=22)	P-value Class II vs Lean	P-Value Class II vs Class I	Class III obese group (n=22)	P-value Class III vs Lean	P-Value Class III vs Class I	P-Value Class III vs Class II
Age (years)	30.14±2.12	31.14±2.46	0.62	31.18±3.22	0.58	1.00	32.05±2.95	0.10	0.69	0.72
Age range (years)	(27-34)	(28-37)		(27-38)			(27-37)			
BMI (kg/m^2^)	23.03±1.08	32.77±1.11^a^	0.000	37.11±1.78^a^^b^	0.000	0.000	41.79±1.93^a^^b^^c^	0.000	0.000	0.000
WC (cm)	83.39±3.89	101.84±4.08^a^	0.000	109.23±5.27^a^^b^	0.000	0.000	117.36±6.22^a^^b^^c^	0.000	0.000	0.000
WC/Height Ratio	0.50±0.02	0.61±0.03^a^	0.000	0.65±0.03^a^^b^	0.000	0.001	0.70±0.04^a^^b^^c^	0.000	0.000	0.000
FBG (mg/dL)	83.14±5.26	90.05 ±5.71^a^	0.000	96.14±4.80^a^^b^	0.000	0.001	104.59±3.71^a^^b^^c^	0.000	0.000	0.000
Insulin (µIU/mL)	8.46±1.67	10.96±1.72^a^	0.000	11.99±1.80^a^	0.000	0.23	14.42±1.96^a^^b^^c^	0.000	0.000	0.000
HOMA-IR	1.73±0.34	2.43 ±0.37^a^	0.000	2.84±0.43^a^^b^	0.000	0.008	3.72±0.50^a^^b^^c^	0.000	0.000	0.000
TC (mg/dL)	145.41±17.20	181.55 ±19.40^a^	0.000	198.09±16.31^a^^b^	0.000	0.02	210.14±20.16^a^^b^	0.000	0.000	0.13
TG mg/dL)	93.27±15.30	129.32 ±18.41^a^	0.000	142.27±17.57^a^	0.000	0.06	160.23±16.80^a^^b^^c^	0.000	0.000	0.004
HDL-C (mg/dL)	57.18±4.17	44.91 ±4.03^a^	0.000	40.14±3.96^a^^b^	0.000	0.000	36.05±2.59^a^^b^^c^	0.000	0.000	0.003
LDL-C (mg/dL)	69.57±17.81	110.77 ±21.37^a^	0.000	129.50±17.73^a^^b^	0.000	0.008	142.05±18.96^a^^b^	0.000	0.000	0.14
IL-6 (pg/mL)	2.27±0.51	5.27 ±0.98^a^	0.000	7.96±1.24^a^^b^	0.000	0.000	10.79±1.49^a^^b^^c^	0.000	0.000	0.000
Clusterin (µg/mL)	33.88±8.23	44.45 ±6.78^a^	0.000	54.29±5.94^a^^b^	0.000	0.000	66.84±7.70^a^^b^^c^	0.000	0.000	0.000
Irisin (ng/mL)	56.25±8.26	79.04±9.55^a^	0.000	86.50±11.12^a^	0.000	0.051	97.15±10.20^a^^b^^c^	0.000	0.000	0.002

Data are represented as mean ± SD and range.

BMI: body mass index; WC: waist circumference; FBG: fasting blood glucose; HOMA-IR: Homeostatic Model Assessment for Insulin Resistance; TC: total cholesterol; TG: triglycerides; HDL-C: high-density lipoprotein cholesterol; LDL-C: low-density lipoprotein cholesterol; IL-6: interleukin-6.

a Significant difference versus lean group (P < 0.05).

b Significant difference versus class I obese group (P < 0.05).

c Significant difference versus class II obese group (P < 0.05).

Data were analyzed by One-way Analysis of Variance (ANOVA) followed by post hock Tukey’s HDS test.

As Compared to class I obese male group, class II obese male group showed significant elevation in the majority of the measured variables (p<0.05) except for HDL-C which showed significant decrease (p=0.000). However, class II obese male group showed non-significant elevation in fasting plasma insulin, TG, and irisin levels as compared to class I obese male group (p>0.05).

When compared to both class I and class II obese male groups, class III obese male group showed significant increase in the majority of the measured parameters (p<0.05) except for HDL-C level which showed significant decline (p=0.000 and p=0.003 respectively). However, class III obese male group showed non-significant elevation in TC and LDL-C levels as compared to class II obese male group (p>0.05).

The anthropometric data and biochemical parameters in the different studied female groups are shown in Table 2. As compared to female lean group, class I obese female group showed significant increase in all measured parameters (p<0.05) except for HDL-C level which showed significant decrease (p=0.000) and fasting insulin level which showed non-significant elevation (p=0.09).

**Table 2:** Anthropometric data and biochemical parameters in the different studied female groups

Parameters	Lean group (n=22)	Class I obese group (n=22)	P-value Class I vs Lean	Class II obese group (n=22)	P-value Class II vs Lean	P-Value Class II vs Class I	Class III obese group (n=22)	P-value Class III vs Lean	P-Value Class III vs Class I	P-Value Class III vs Class II
Age (years)	29.95±2.01	30.09±2.69	1.00	30.77±3.05	0.75	0.84	31.68±2.93	0.16	0.21	0.68
Age range (years)	(27-34)	(24-35)		(24-36)			(27-36)			
BMI (kg/m^2^)	23.74±1.41	32.42±1.47^a^	0.000	36.62±1.42^a^^b^	0.000	0.000	41.37±1.48^a^^b^^c^	0.000	0.000	0.000
WC (cm)	75.45±4.31	92.50±3.07 a	0.000	98.41±4.15^a^^b^	0.000	0.001	107.41±5.12^a^^b^^c^	0.000	0.000	0.000
WC/Height Ratio	0.46±0.03	0.56±0.02 a	0.000	0.59±0.03 ab	0.000	0.003	0.65±0.04^a^^b^^c^	0.000	0.000	0.000
FBG (mg/dL)	83.05±4.13	87.14±5.73^a^	0.04	91.14±4.54^a^^b^	0.000	0.044	97.09±5.51^a^^b^^c^	0.000	0.000	0.001
Insulin (µIU/mL)	8.06±1.95	9.32±1.89	0.09	10.48±1.40^a^	0.000	0.134	12.32±1.73^a^^b^^c^	0.000	0.000	0.004
HOMA-IR	1.65±0.39	2.01±0.44^a^	0.031	2.36±0.38^a^^b^	0.000	0.032	2.96±0.47^a^^b^^c^	0.000	0.000	0.000
TC (mg/dL)	142.95±13.78	171.50±17.36^a^	0.000	183.50±17.61^a^	0.000	0.10	199.32±19.02^a^^b^^c^	0.000	0.000	0.02
TG mg/dL)	90.55±19.29	113.18±15.63^a^	0.000	124.55±16.25^a^	0.000	0.14	142.05±17.71^a^^b^^c^	0.000	0.000	0.006
HDL-C (mg/dL)	58.14±4.09	49.55 ±3.46^a^	0.000	45.27±4.22^a^^b^	0.000	0.002	40.73±3.06^a^^b^^c^	0.000	0.000	0.001
LDL-C (mg/dL)	66.71±15.27	99.32±17.43^a^	0.000	113.32±19.01^a^	0.000	0.052	130.18±19.25^a^^b^^c^	0.000	0.000	0.012
IL-6 (pg/mL)	2.13±0.53	3.37 ±0.85^a^	0.004	5.86±1.12^a^^b^	0.000	0.000	8.32±1.80^a^^b^^c^	0.000	0.000	0.000
Clusterin (µg/mL)	29.58±6.28	40.37±5.40^a^	0.000	45.09±7.56^a^	0.000	0.091	57.37±6.99^a^^b^^c^	0.000	0.000	0.000
Irisin (ng/mL)	53.78±7.20	64.11±8.69^a^	0.003	77.03 ±9.69^a^^b^	0.000	0.000	91.93 ±11.43^a^^b^^c^	0.000	0.000	0.000

Data are represented as mean ± SD and range.

BMI: body mass index; WC: waist circumference; FBG: fasting blood glucose; HOMA-IR: Homeostatic Model Assessment for Insulin Resistance; TC: total cholesterol; TG: triglycerides; HDL-C: high-density lipoprotein cholesterol; LDL-C: low-density lipoprotein cholesterol; IL-6: interleukin-6.

a Significant difference versus lean group (P<0.05).

b Significant difference versus class I obese group (P<0.05).

c Significant difference versus class II obese group (P<0.05).

Data were analyzed by One-way Analysis of Variance (ANOVA) followed by post hock Tukey’s HDS test.

When compared to lean female group, both class II and class III obese female groups showed significant increase in all measured variables (p=0.000) except for HDL-C level which showed significant decrease (p=0.000).

As compared to class I obese female group, class II obese female group showed significant increase in the majority of the measured parameters (p<0.05) except for HDL-C level which showed significant decrease (p=0.002). However, class II obese female group showed non-significant elevation in the fasting insulin, TC, triglycerides and LDL-C levels when compared to class I obese female group (p>0.05).

When compared to class I obese female group, class III obese female group showed significant elevation in all measured variables (p=0.000) except for HDL-C level which showed significant decrease (p=0.000).

As compared to class II obese female group, class III obese female group showed significant increase in all measured variables (p<0.05) except for HDL-C level which showed significant decrease (p=0.001).

The anthropometric data and biological parameters in the different studied male and female groups are shown in Table 3. There was non-significant difference between lean male group and lean female group in all variables except for WC and WC/Height ratio which showed significant elevation in lean male group as compared to lean female group (p=0.000 and p=0.000 respectively). As compared to class I obese female group, class I obese male group showed significantly higher levels in the majority of the measured variables (p<0.05) except for HDL-C level which showed significant decrease (p=0.000). However, there were non-significant differences between class I obese male group and class I obese female group in BMI, TC and LDL-C (p>0.05). As compared to class II and class III obese female groups, class II and class III obese male groups showed significant increase in the majority of the measured variables (p<0.05) except for HDL-C level which showed significant decrease (p=0.000 and p=0.000 respectively). Non-significant elevations in triglycerides and irisin levels were observed when comparing class III obese male group with class III obese female group (p>0.05).

**Table 3:** Anthropometric data and biochemical parameters in the different studied both male and female groups

Variables	Male Lean group (n=22)	Female Lean group (n=22)	P-value	Male Class I obese group (n=22)	Female Class I obese group (n=22)	P-value	Male Class II obese group (n=22)	Female Class II obese group (n=22)	P-value	Male Class III obese group (n=22)	Female Class III obese group (n=22)	P-value
Age (years)	30.14±2.12	29.95±2.01	0.77	31.14±2.46	30.09±2.69	0.19	31.18±3.22	30.77±3.05	0.67	32.05±2.95	31.68±2.93	0.68
BMI (kg/m^2^)	23.03±1.08	23.74±1.41	0.07	32.77 ±1.11	32.42±1.47	0.37	37.11±1.78	36.62±1.42	0.31	41.79±1.93	41.37±1.48	0.42
WC (cm)	83.39±3.89^a^	75.45±4.31	0.000	101.84±4.08^b^	92.50±3.07	0.000	109.23±5.27^c^	98.41±4.15	0.000	117.36±6.22^d^	107.41±5.12	0.000
WC/Height	0.50±0.02^a^	0.46±0.03	0.000	0.61±0.03^b^	0.56±0.02	0.000	0.65±0.03^c^	0.59±0.03	0.000	0.70±0.04^d^	0.65±0.04	0.000
FBG (mg/dL)	83.14±5.26	83.05±4.13	0.95	90.05±5.71^b^	87.14±5.73	0.01	96.14±4.80^c^	91.14±4.54	0.001	104.59±3.71^d^	97.09±5.51	0.000
Insulin (µIU/mL)	8.46±1.67	8.06±1.95	0.47	10.96±1.72^b^	9.32±1.89	0.005	11.99±1.80^c^	10.48±1.40	0.003	14.42±1.96^d^	12.32±1.73	0.001
HOMA-IR	1.73±0.34	1.65±0.39	0.45	2.43±0.37^b^	2.01±0.44	0.001	2.84±0.43^c^	2.36±0.38	0.000	3.72±0.50^d^	2.96±0.47	0.000
TC (mg/dL)	145.41±17.2	142.95±13.78	0.60	181.55±19.40	171.50±17.36	0.08	198.09±16.31^c^	183.50±17.61	0.007	210.14±20.16	199.32±19.02	0.07
TG (mg/dL)	93.27±15.30	90.55±19.29	0.61	129.32±18.41^b^	113.18±15.63	0.003	142.27±17.57^c^	124.55±16.25	0.001	160.23±16.8^d^	142.05±17.71	0.001
HDL-C (mg/dL)	57.18±4.17	58.14±4.09	0.45	44.91±4.03^b^	49.55±3.46	0.000	40.14±3.96^c^	45.27±4.22	0.000	36.05±2.59^d^	40.73±3.06	0.000
LDL-C (mg/dL)	69.57±17.81	66.71±15.27	0.57	110.77±21.37	99.32±17.43	0.06	129.50±17.73^c^	113.32±19.01	0.006	142.05±18.96^d^	130.18±19.25	0.046
IL-6 (pg/mL)	2.27±0.51	2.13±0.53	0.35	5.27±0.98^b^	3.37±0.85	0.000	7.96±1.24^c^	5.86±1.12	0.000	10.79±1.49^d^	8.32±1.80	0.000
Clusterin (µg/mL)	33.88±8.23	29.58±6.28	0.06	44.45±6.78^b^	40.37±5.40	0.03	54.29±5.94^c^	45.09±7.56	0.000	66.84±7.70^d^	57.37±6.99	0.000
Irisin (ng/mL)	56.25±8.26	53.78±7.20	0.17	79.04±9.55^b^	64.11±8.69	0.000	86.50±11.12^c^	77.03±9.69	0.004	97.15±10.20	91.93±11.43	0.12

Data are represented as mean ± SD.

BMI: body mass index; WC: waist circumference; FBG: fasting blood glucose; HOMA-IR: Homeostatic Model Assessment for Insulin Resistance; TC: total cholesterol; TG: triglycerides HDL-C: high-density lipoprotein cholesterol; LDL-C: low-density lipoprotein cholesterol; IL-6: interleukin-6.

a Significant difference lean female versus lean male group (P<0.05).

b Significant difference class I obese female group versus class I obese male group (P<0.05).

c Significant difference class II obese female group versus class II obese male group (P<0.05).

d Significant difference class III obese female group versus class III obese male group (P<0.05).

Data were analyzed by independent t-test.

**Figure 2:**
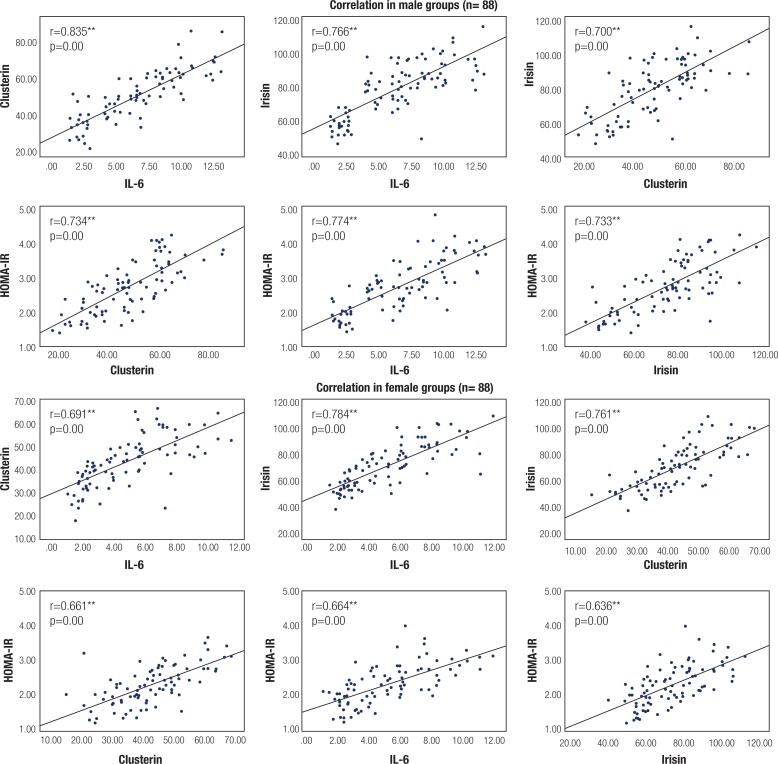
Pearson correlation of HOMA-IR, IL-6, Clusterin and Irisin in both sexes.

Pearson correlation analysis between the measured variables is shown in Table 4 and Figure 2. In both sexes, circulating IL-6, clusterin and irisin levels showed significant positive association with BMI, fasting blood glucose level, fasting insulin level, HOMA-IR and lipid profile (TC, TG, LDL-C) except for HDL-C which showed significant negative correlation. For both sexes, circulating irisin exhibited significant positive correlation with both clusterin and IL-6. Also, circulating IL-6 was significantly and positively correlated with clusterin. Furthermore, in both sexes, WC was significantly and positively correlated with BMI, fasting blood glucose, fasting insulin, HOMA-IR, IL-6, irisin, clusterin and lipid panel except for HDL-C which showed significant negative correlation.

**Table 4:** Pearson correlation of IL-6, Clusterin and Irisin versus BMI, FBG, Insulin, HOMA-IR and Lipid profile in both sexes

	IL-6		Irisin		Clusterin		WC	
	Male	Female	Male	Female	Male	Female	Male	Female
	r	r	r	r	r	r	r	r
BMI	0.901**	0.821**	0.837**	0.806**	0.842**	0.808**	0.975**	0.968**
FBG	0.819**	0.678**	0.696**	0.644**	0.750**	0.595**	0.765**	0.654**
Insulin	0.665**	0.579**	0.668**	0.557**	0.647**	0.600**	0.686**	0.604**
HOMA-IR	0.774**	0.664**	0.733**	0.636**	0.734**	0.661**	0.766**	0.671**
TC	0.754**	0.685**	0.704**	0.601**	0.641**	0.543**	0.726**	0.725**
TG	0.771**	0.596**	0.708**	0.594**	0.668**	0.608**	0.743**	0.703**
HDL-C	-0.856**	-0.729**	-0.795**	-0.658**	-0.769**	-0.737**	-0.815**	-0.802**
LDL-C	0.774**	0.706**	0.723**	0.612**	0.667**	0.577**	0.743**	0.742**
IL-6			0.766**	0.784**	0.835**	0.691**	0.858**	0.782**
Clusterin	0.835**	0.691**	0.700**	0.761**			0.811**	0.748**
Irisin							0.818**	0.777**

BMI body mass index; FBG fasting blood glucose; HOMA-IR: Homeostatic Model Assessment for Insulin Resistance; TC: total cholesterol; TG: triglycerides; HDL-C: high-density lipoprotein cholesterol; LDL-C: low-density lipoprotein cholesterol; IL-6: interleukin-6; WC: waist circumference.

* Correlation is significant at the 0.05 level (2-tailed).

** Correlation is significant at the 0.01 level (2-tailed).

## DISCUSSION

This study aimed at evaluating the circulating levels of IL-6, clusterin, and irisin in obese subjects of both sexes who had different grades of obesity and examining their sexual dimorphism and their association with insulin resistance. Briefly, our data revealed that, obesity in both sexes, especially in males was associated with high levels of circulating IL-6, clusterin and irisin and worsened the metabolic pattern.

During the current study, IL-6 level was significantly elevated in obese subjects of both sexes as compared to lean subjects. This elevation in IL-6 levels was significantly and positively associated with BMI. Our former result is in accordance with the previously reported results postulated that, there is a positive correlation between IL-6 and BMI (24). Regarding gender difference, obese men showed significantly higher IL-6 levels than obese women. This result may be explained on the basis that, the female sex hormone estrogen could inhibit IL-6 production and down-regulate IL-6 receptor (25). This result seems in parallel with a previously reported finding demonstrated higher IL-6 level in males than females (26). However, our result seems in contradiction with other previous findings demonstrate the reverse (27). For both sexes, we observed presence of significant positive correlation between circulating IL-6 level and lipid panel except for HDL-C which exhibited significant negative correlation. IL-6 was reported to have negative impact on lipid metabolism through triglyceride release and lipoprotein lipase down-regulation (4). In addition, IL-6 was reported to negatively affect adiponectin regulation and decreases adiponectin level. Low adiponectin level is formerly known to impair insulin action in adipose tissue with subsequent elevated rates of lipolysis, increased free fatty acid release and decreased HDL-C biosynthesis (28). For both sexes, we observed presence of significant positive association between IL-6 and HOMA-IR index. It was postulated that, elevated IL-6 level is associated with insulin resistance and the development of types 2 diabetes mellitus through its suppressive effect on glucose transporter-4 and insulin receptor substrate-1 expression (8,29).

In the present study, plasma clusterin level was significantly higher in obese subjects of both sexes as compared to lean persons and its level tended to be increased with the increase in adiposity. This result may be attributed to the notion that, clusterin is a sensitive biomarker of oxidative stress which reflects the pro-inflammatory and oxidative stress states associated with the increase in adiposity (1,3,11). In this context, elevated plasma clusterin level may represent a defense mechanism to attenuate obesity related oxidative stress and inflammation. For both sexes, we observed presence of significant positive correlation between plasma clusterin level and BMI. Our result is in agreement with a previous finding postulated presence of positive association between circulating clusterin and BMI (11), but it seems inconsistent with other previous finding (17). Regarding gender difference, obese males showed significantly higher plasma clusterin level than obese females. This finding seems in accordance with previously reported findings (11). We cannot attribute this sex bias in circulating clusterin level to the effect of smoking or to the effects of sex hormones since our participants were non-smokers and the effects of both male and female sex hormones on circulating clusterin level were reported to be inconsistent (11,21). In this context, increased clusterin level in obese males than obese females may be related to significantly higher inflammatory state (IL-6 level) in obese men than obese women since circulating clusterin was reported to be remarkably elevated after exposure to inflammatory cytokines (15). For both sexes, we observed presence of significant positive correlation between circulating clusterin and lipid panel except for HDL-C which showed significant negative association. Clusterin was reported to be overproduced from the fat cells of obese subjects and its level was linked to harmful cholesterol levels (3). In addition, our result comes in parallel with some previously reported findings demonstrated presence of positive association between circulating clusterin with total cholesterol and LDL-C and its negative association with HDL-C (30). For both sexes, we observed presence of significant positive association between circulating clusterin and HOMA-IR, a result seems in agreement with previous findings (3). This former result was attributed to the ability of clusterin to attenuate insulin signaling and to its ability to stimulate hepatic gluconeogenesis (3). Also, for both genders, we observed presence of significant positive association between circulating clusterin and IL-6. This result is in consonance with previously reported findings demonstrated that, clusterin may reflect the pro-inflammatory state associated with adiposity and there was a positive association between circulating clusterin and systemic inflammation (11). Furthermore, it was postulated that, circulating clusterin level was remarkably elevated after exposure to inflammatory cytokines (15).

During the current study, irisin level was significantly higher in obese subjects of both sexes as compared to lean subjects. In consonance with our result, circulating irisin was reported to be higher in obese individuals than non-obese control which was justified as a state of possible irisin resistance (18). However, our result seems in contradiction with a previously reported finding postulated that, irisin level tended to be decreased with the increase in adiposity (19). These conflicting results may be related to ethnicity, genetic difference, insulin resistance, pre-diabetes and diabetes that can influence specific hormones including irisin (18,31). For both sexes, we observed presence of significant positive correlation between circulating irisin and BMI. Our result comes in agreement with a former study demonstrated presence of positive association between irisin and anthropometric indices in obese subjects (32). However, our result seems in contradiction with a former study demonstrated presence of negative association (2). Regarding gender difference, irisin level was significantly higher in obese males than obese females except for class III obesity. This result seems in matching with Murawska-Cialowicz and cols., 2015 who reported that, irisin level was higher in men than women (22). However, our result is in contradiction with a previous finding showed lower irisin levels in obese men than obese women (23). For both sexes, we observed presence of significant positive association between irisin and all lipid panels except for HDL-C which showed significant negative association. Therefore, our result may propose that, higher irisin level may be related to an atherogenic lipid panel. Our result is in agreement with previously reported findings postulated the existence of positive association of irisin with total cholesterol and triglycerides among non-diabetic subjects (31). In contrast, our result seems in disagreement with the notion that, favorable lipid profile is associated with elevated irisin concentration (33). For both sexes, we noted presence of significant positive association between circulating irisin and HOMA-IR. This result may be attributed to a state of possible irisin resistance which could potentially modulate insulin sensitivity (34). Similar to our results, some authors reported the existence of positive association between irisin and HOMA-IR (20). However, our result seems in contradiction with a previously reported finding demonstrated presence of negative association between irisin and insulin resistance (2). For both sexes, we noted presence of significant positive correlation between circulating irisin and IL-6. In agreement with our finding, some authors reported that, irisin secretion tended to be increased during inflammatory state (35). In contradiction with our finding, it was postulated that, irisin has anti-inflammatory property and the treatment with irisin resulted in a decrease in the expression of pro-inflammatory cytokines (36). Also, we observed presence of significant positive association between irisin and clusterin. This finding may be attributed to a compensatory defense mechanism of increased circulating irisin level to neutralize obesity associated oxidative stress through its antioxidant activity (37). However, the role of irisin in obesity is still at need for further investigations.

In the present study, obese subjects of both genders showed more atherogenic lipid profile and more insulin resistance as compared to lean subjects. Additionally, obese men showed more atherogenic lipid panel and higher HOMA-IR index than obese women. These results seem in agreement with previously reported findings postulated that, obese subjects showed elevated fasting plasma glucose and triglyceride levels and low HDL-C level with significant gender differences (38). The higher HOMA-IR index observed in obese males than obese females during the current study may be attributed to higher state of possible irisin resistance, higher inflammatory state (higher IL-6 level) and a possible increase in oxidative stress state (higher clusterin level) which may in turn contribute to insulin resistance (8,39).

However, BMI remains the gold standard for identifying patients at higher risk for adiposity related adverse health outcomes, waist circumference (WC), which defines abdominal obesity, is usually associated with cardiometabolic diseases risk (40). During the current study, WC tended to be increased with adiposity and there was a significant positive assciation between WC and BMI, a result comes in agreement with a previously reported finding (41). Furthermore, male group showed significantly higher WC than female group, a result seems in accordanace with some former findings (41,42). For both sexes, we observed presence of significant positive correlation of WC with HOMA-IR and fasting insulin level which seems in consonance with a previously reported finding (41). WC was associated with an atherogenic lipid profile which comes in matching with some former findings (43,44). Finally, WC was significantly and positively associated with IL-6, clusterin, and irisin. Our later result is in parallel with previously reported findings (11,42,45). In sum, WC was positively associated with insulin resistance, dyslipidemia, inflammation and clusterin (the indirect marker of oxidative stress). Therefore, obese subjects with increased WC, especially men may have an increased risk for cardiometabolic diseases.

In conclusion, in obese subjects of both sexes, circulating IL-6, clusterin and irisin levels were significantly elevated and positively correlated with BMI. Regarding gender difference, obese men had more atherogenic lipid profile, higher state of possible irisin resistance (except for class III obesity), higher inflammatory state, elevated clusterin levels which would reflect a possible increase in oxidative stress state, more insulin resistance and higher risk for cardiometabolic diseases than obese females. Furthermore, in both sexes, circulating IL-6, clusterin and irisin levels were significantly and positively associated with HOMA-IR index. In this context, circulating IL-6, clusterin and irisin may represent possible therapeutic targets for insulin resistance in obese subjects.
